# Relationships between static foot alignment and dynamic plantar loads in runners with acute and chronic stages of plantar fasciitis: a cross-sectional study

**DOI:** 10.1590/bjpt-rbf.2014.0136

**Published:** 2016-01-19

**Authors:** Ana P. Ribeiro, Isabel C. N. Sacco, Roberto C. Dinato, Silvia M. A. João

**Affiliations:** 1 Departamento de Fisioterapia, Fonoaudiologia e Terapia Ocupacional, Faculdade de Medicina, Universidade de São Paulo (USP), São Paulo, SP, Brazil; 2 Pós-Graduação em Ciências da Saúde, Departamento de Fisioterapia, Faculdade de Medicina, Universidade de Santo Amaro (Unisa), São Paulo, SP, Brazil

**Keywords:** plantar fasciitis, foot, plantar arch, physical therapy, overload, running

## Abstract

**BACKGROUND:**

The risk factors for the development of plantar fasciitis (PF) have been associated with the medial longitudinal arch (MLA), rearfoot alignment and calcaneal overload. However, the relationships between the biomechanical variables have yet to be determined.

**OBJECTIVE:**

The goal of this study was to investigate the relationships between the MLA, rearfoot alignment, and dynamic plantar loads in runners with unilateral PF in acute and chronic phases.

**METHOD:**

Cross-sectional study which thirty-five runners with unilateral PF were evaluated: 20 in the acute phase (with pain) and 15 with previous chronic PF (without pain). The MLA index and rearfoot alignment were calculated using digital images. The contact area, maximum force, peak pressure, and force-time integral over three plantar areas were acquired with Pedar X insoles while running at 12 km/h, and the loading rates were calculated from the vertical forces.

**RESULTS:**

The multiple regression analyses indicated that both the force-time integral (*R* ^2^=0.15 for acute phase PF; *R* ^2^=0.17 for chronic PF) and maximum force (*R* ^2^=0.35 for chronic PF) over the forefoot were predicted by an elevated MLA index. The rearfoot valgus alignment predicted the maximum force over the rearfoot in both PF groups: acute (*R* ^2^=0.18) and chronic (*R* ^2^=0.45). The rearfoot valgus alignment also predicted higher loading rates in the PF groups: acute (*R* ^2^=0.19) and chronic (*R* ^2^=0.40).

**CONCLUSION:**

The MLA index and the rearfoot alignment were good predictors of plantar loads over the forefoot and rearfoot areas in runners with PF. However, rearfoot valgus was demonstrated to be an important clinical measure, since it was able to predict the maximum force and both loading rates over the rearfoot.

## Introduction

Foot types and repetitive plantar loads have been commonly associated with lower limb injuries, especially running-related injuries^1^, such as medial tibial stress syndrome^2^, patellofemoral pain syndrome^3^, and plantar fasciitis (PF)^4^^-^ 6. Among them, PF is a musculoskeletal disorder characterized by pain at the plantar fascia insertion point^7^. PF is considered to be the third most prevalent injury in runners^8^^-^ 10. Despite its high prevalence, knowledge about its pathogenesis is still limited^7^. However, specific intrinsic and extrinsic risk factors related to the foot-ankle structures have been explored in the literature^11^. The main intrinsic factors for the development of PF in runners have been explained as foot-type changes^12^, rearfoot valgus posture^4^^,^ 13 ^,^ 14, and elevated plantar arch structures^5^^,^ 8 ^,^ 13. Understanding the foot structure has been the main focus of clinicians to prevent injuries in runners, helping them to choose the correct footwear and providing the appropriate interventions^15^. These are directed towards improving the synaptic tactile afferents from the fascia and the motor neurons supplying the leg muscles^1^^,^ 16.

Studies of runners with PF have shown that changes in the medial longitudinal arch (MLA) geometry (higher^5^^,^ 6 or lower^4^) and the presence of pain contribute to increasing plantar loads^4^^,^ 13. Di Caprio et al.^5^ described a higher arch as a great predictor of PF in runners, because an elevated MLA could induce greater stiffness of the plantar fascia, resulting in less flexible tissue^5^. This could result in the inefficient capacity to dissipate foot impact forces, with greater mechanical stress on the calcaneus^17^, interfering with the dynamic foot function^18^.

Elevated MLAs in healthy runners have been associated with higher vertical loading rates^19^^,^ 20 and peak pressures over the rearfoot while running^20^. However, some studies have reported a lack of association between static and dynamic elevated MLAs and the loading rates or peak pressures during running^21^^,^ 22. Recently, the combination of MLAs and rearfoot eversion angles were described as good predictors of the pressure-time integral over the rearfoot and midfoot in healthy runners^23^. The increased rearfoot pronation associated with a lower MLA could also result in greater plantar loads over the calcaneal medial area^24^^-^ 27, which, in turn, induces greater stretch in the plantar fascia^10^^,^ 17. A valgus alignment of the calcaneus or pronated foot posture significantly increases the likelihood of generalized foot pain^18^.

The microtrauma and microtearing potentially caused by an elevated MLA and a valgus rearfoot are the primary mechanisms of PF, resulting in the inflammation characteristic of the acute phase^28^^,^ 29. The progression of PF can lead to a symptom remission phase, with the evolution of fragmentation and degeneration of the plantar fascia, characterizing the chronic phase^29^^,^ 30. Previous gait studies in individuals with PF determined that the pain stimulus promoted changes in foot roll-over patterns, thus causing load reductions in the rearfoot and load increases in the midfoot^31^, forefoot^32^, and toes^32^^,^ 33, possibly due to the protective mechanisms of pain. A deep comprehension of the changes in plantar pressure associated with static foot posture may provide useful information for the prescription or design of interventions, such as orthotics or motion-control shoes for runners with PF.

The purpose of this study was to investigate the relationships between the MLA index and rearfoot alignment with plantar loads in runners with PF in the acute and chronic phases. We hypothesized that (1) an elevated MLA will predict lower plantar loads over the rearfoot in runners with acute PF, due to an antalgic mechanism, and higher loads in runners with chronic PF; and (2) a valgus rearfoot alignment would predict higher plantar loads and loading rates over the rearfoot in both groups of runners.

## Method

### Participants

This cross-sectional study examines the relationship between PF and foot alignment. For this, thirty-five runners of both sexes with diagnoses of unilateral PF were recruited from the Rehabilitation Center of Sport Rheumatology at Hospital Universitário de São Paulo, Brazil. The mean running speed of their last 10 km competition was 11.5±0.4 km/h. The inclusion criteria were: runners must have run at least 20 km weekly for at least one year, be experienced in long-distance competitions, have a regular rearfoot strike pattern, and have a diagnosis of unilateral PF confirmed by a clinical examination. The exclusion criteria were a history of previous surgery in the lower limbs, traumas or fractures of the lower limbs in the previous six months, leg length discrepancies, or other musculoskeletal disorders such as neuropathies, rheumatoid arthritis, or calcaneal spurs. This protocol was approved by the Human Research Ethics Committee of the School of Medicine of Universidade de São Paulo (USP), São Paulo, SP, Brazil (number: 384/10; title: Support standard and impact of the feet with the ground during the running of runners with history and symptoms of plantar fasciitis and its relationship to the medial longitudinal arch). All participants provided written consent.

All of the runners had diagnoses of unilateral PF confirmed by clinical examination and ultrasound images. Twenty runners were included in the acute PF group; they had acute inflammation and perifascial fluid detected in the ultrasound images combined with pain symptoms in the calcaneus for more than four months (mean of 4.0±2.0 months), with mean intensity of 8.1 cm (measured by a 0-10 visual analog scale). The pain was present during palpation of the plantar fascia after waking up in the morning, while remaining in the standing position, when taking the first few steps, while sitting for long periods of time, and after physical activity^32^^,^ 33.

Fifteen runners had previous chronic stages of PF with a mean time since the first diagnosis of 1.5±3.0 years and cycles of remission within the period between the diagnosis and the biomechanical evaluation. In this group, we only included runners with unilateral PF who showed plantar fascial thickness, fragmentation, and degeneration in the ultrasound, but no signs of acute inflammatory processes or pain complaints over the previous two months^30^.

Both groups (acute and chronic) demonstrated similar anthropometric characteristics and running practices (Table 1). In addition, all of the runners with PF were asked about any interventions previously used to treat this injury. The most frequent clinical interventions described by the runners were: physical therapy combined with medication (38%); insoles (27%); medication alone (21%); and other interventions such as acupuncture and manual therapy (14%).

**Table 1:** - Descriptive statistics (mean ± standard deviation) and comparisons between acute plantar fasciitis (FP) and chronic plantar fasciitis (PF) regarding their demographic, anthropometric, and running practice characteristics.

Variables	Acute PF (n=20)	Sex (Acute PF) (n=13 M; 7 F)	Chronic PF (n=15)	Sex (Chronic PF) (n=10 M; 5 F)	p*
Age (years)	42.8±9.3	M (46.1±8.3) F (44.5±9.0)	38.3±7.3	M (37.8±6.5) F (34.6±4.3)	0.126
Body mass (Kg)	70.1±14.5	M (77.1±8.8) F (59.2±9.5)	72.3±10.0	M (75.4±8.3) F (60.0±9.8)	0.641
Height (m)	1.70±9.9	M (1.74±4.7) F (158.7±6.1)	1.76±7.8	M (1.79±5.7) F (1.6±3.6)	0.224
Body mass index (Kg/m^2^)	24.6±2.7	M (25.3±1.8) F (23.4±1.9)	23.0±2.0	M (23.3±1.8) F (22.3±2.2)	0.090
Training volume (km/week)	41.0±9.0	M (42.8±7.7) F (40.1±4.6)	45.0±10.0	M (46.4±8.1) F (40.0±3.4)	0.147
Practice time (years)	8.0±5.5	M (9.7±7.0) F (5.5±1.7)	6.2±5.0	M (7.2±6.1) F (6.0±1.4)	0.382

Acronym: M for male; F for female; PF: Plantar Fasciitis. *Calculated by ANOVAs one-way between groups (Acute and Chronic of PF), post-hoc: Tukey..

### Static measurements of the structures of the ankle and foot

#### Assessment of the frontal alignment of the rearfoot (calcaneal tendon)

**Figure 1:**
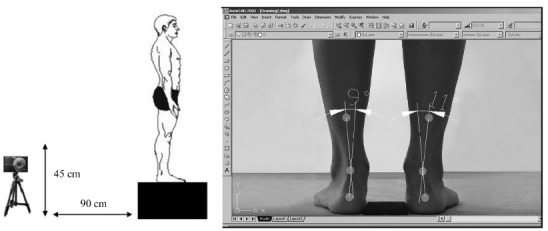
- Position of individual and digital camera to capture digital image of rearfoot angle and measurement of the frontal alignment of the rearfoot in AutoCAD software.

To evaluate the alignment of the rearfoot in the posterior view of the frontal plane, the runners stood over a 45 cm platform, keeping their feet 7.5 cm apart. With a dermatographic pen and 9 mm white markers, the following anatomical points were identified on the inferoposterior regions of both legs: 1) the posterior calcaneal tuberosity; 2) the second point above the center of the calcaneus; and 3) the lower third of the leg^13^^,^ 34 ^,^ 35 (Figure 1). The center of each marker in the medial-lateral axis was obtained with a digital caliper that was used to measure the distances between the two symmetrically opposing sides with a ruler^13^. The images were then obtained with a digital camera positioned anterior and perpendicular to the subjects at a distance of 90 cm and at a height of 45 cm. The images obtained (with a minimum size of 768 pixels) were analyzed on a 96-ppi screen, due the good inter-examiner reliability for the photogrammetric measurements of the rearfoot static angles^35^ (Figure 1).

AutoCAD software 2005^(r)^ was used to quantify the alignment of the rearfoot. For this, a line was drawn from the first marker (posterior calcaneal tuberosity) to the second marker (calcaneal center). A second straight line was then drawn, which originated from the lower third of the leg marker and passed through the second marker (calcaneal center)^13^^,^ 35 (Figure 1). The intersection of the extensions of both straight lines resulted in angles, which were classified as normal foot (0° to 5°), varus (< 0°), or valgus (> 5°)^34^.

### Assessment of the medial longitudinal arch (MLA)

**Figure 2:**
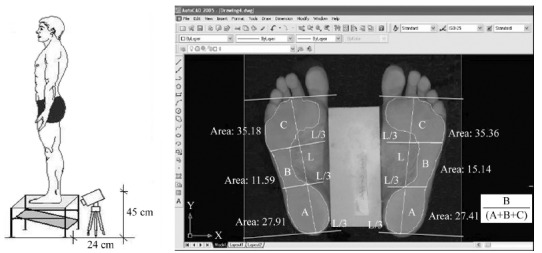
- Image obtained by podoscope (A) and illustration of the areas of the feet to calculate the longitudinal plantar arch index (MLA), where L: vertical line and areas A: rearfoot, B: midfoot and C: forefoot (B).

The footprint was acquired using a Carci^(r)^ podoscope. For the barefoot assessments, the subjects were positioned on the podoscope with 7.5 cm of ethylene vinyl acetate (EVA) placed between the feet. The footprint image was obtained with a digital camera, which was placed in front of the podoscope at a distance of 24 cm and a height of 45 cm (Figure 2). The EVA measurement was taken as a reference for the AutoCAD software 2005^(r)^ image scale. In AutoCAD, a vertical line (L) was drawn from the second metatarsus to the center of the calcaneus. Then, the L line was divided into three parts for the delineation of the forefoot, midfoot, and rearfoot areas^13^^,^ 34.

To classify the MLA, the Arch Index^36^ was calculated in the footprint image corresponding to the foot injured by PF. This index is the result of the ratio between the midfoot area and the total area of the foot (Figure 2). Index values between 0.22 and 0.25 correspond to a normal MLA, values smaller than 0.21 correspond to a cavus MLA, and values greater than 0.26 were classified a planus MLA. Footprint analyses via digital imaging were chosen because of the advantages of having the reliability and validity previously confirmed^37^^,^ 38.

### Procedures and instruments for the assessment of plantar loads while running

The plantar pressure distribution while running was obtained using the Pedar X system (Novel, Munich, Germany) at 100 Hz. All of the runners wore standard athletic shoes, which were considered to have neutral support (RAINHA SYSTEM, RAINHA, Alpargatas, São Paulo, Brazil). The shoe characteristics included a midsole made up of ethylene vinyl acetate (EVA, with compression set at 56%, hardness: 57 Asker C, and density = 0.21 g/cm^3^) throughout the entire sole of the shoe. The insoles were placed between the socks and the shoe, and were connected to equipment inside a backpack (about 1.5 Kg).

After a period of adaptation with the shoes, insoles, and backpack, the runners ran a distance of 40 meters on a smooth and regular asphalt track in good conditions at 12 km/h. The speed of the intermediate 20 meters of the track was controlled by two evaluators using a digital chronometer^6^. Two observers used a digital stopwatch to control the speed simultaneously, and the inter-examiner reliability of the speed measurements was calculated using Intra-Class Correlation Coefficients (ICCs). The inter-examiner reliability was excellent (ICC_2,1_=0.96; 95% CI=0.88-0.73), with a standard error of prediction of 0.04.

A mean value of 30 steps per subject was used for statistical purposes, and the variables were calculated using a MATLAB function: contact area (cm), maximum force (N), force-time integral (N.s), and peak pressure (kPa) over the three plantar areas of the rearfoot (30% of the foot length), midfoot (30% of the foot length), and forefoot and toes (40% of the foot length)^6^. Two plantar loading rates were calculated from the vertical force: 1) loading rate of 80% [BW.s^-^ 1], defined as the force rate between 20 and 80% of the first peak, and 2) loading rate of 100% [BW.s^-^ 1], as determined by the force rate between 0 and 100% of the first peak. All of the force variables were normalized by the body weight (BW).

### Statistical analysis

The sample size calculation of the 35 runners with unilateral plantar fasciitis, based upon the maximal force variable, was carried out using G-Power 3.0 software, considering a moderate effect size (*F* =0.25), a statistical power of 80%, and a significance level of 5%. Since all of the outcome variables showed normal distributions (Shapiro-Wilk's test), ANOVAs followed by Tukey's post-hoc tests were used to compare the groups.

To verify our hypothesis that the MLA and rearfoot alignment variables could predict the plantar loads on the foot, we first checked the correlations between these biomechanical variables. Pearson's correlation coefficients were calculated to investigate the relationships between the MLA index and rearfoot alignment and the 20 dependent biomechanical variables: 18 plantar pressure variables over the rearfoot, midfoot, and forefoot (maximum force, force-time integral, maximum mean pressure, peak pressure, pressure-time integral, and contact area), and two loading rates (20 to 80% and 0 to 100% of the first peak [BW.s^-^ 1]).

Forward step-wise multiple regression analyses were used to predict the biomechanical dependent variables (plantar pressure and loading rates) via the MLA index and rearfoot alignment. The biomechanical dependent variables were sequentially included in the model in three consecutive blocks: variables of contact area, force, and pressure. The 20 biomechanical variables were reduced, and only those whose correlation coefficients were higher than 0.20 were entered into the model. For all of the analyses, we adopted p<0.05.

The Pearson correlation analyses between the 20 dependent variables and the MLA index and rearfoot alignment resulted in 11 variables of interest for the regression analyses. The force-time integral, maximum force, and contact area over the rearfoot, midfoot, and forefoot, as well as the loading rates (20 to 80% and 0 to 100%), were included in the model. Nine variables were removed from the model due to low correlation coefficients (r<0.20): maximum mean pressure, peak pressure, and pressure-time integral over the foot areas.

To analyze the intra-rater reliability of the MLA index and rearfoot alignment, the measurements were obtained by the same examiner in two evaluation moments, with a one-week interval, and the intraclass correlation coefficients (ICC_3,1_) were calculated. To investigate the inter-rater reliability, ICC_2,1_ were calculated using the data collected during the first week by two independent examiners^39^. The intra- and inter-rater reliability analyses for the anatomical marker data were performed only by the first examiner, following previously recommended procedures^37^^,^ 38. Both evaluations were performed for both of the PF groups, without separating the phase of the injury.

In order to determine the systematic error of the MLA and rearfoot angle measurements for each examiner (intra- and inter-examiner reliability), the standard error of measurement (SEM) and standard error of prediction (SEP) were calculated. The intra-examiner reliability SEM was calculated as the ratio between the variability (standard deviation, SD) of the mean difference scores between the two repeated measurements, and the √2. The inter-examiner reliability SEP was calculated as: the product of the variability (SD) of the measurements obtained by each examiner and the √1-ICC2^35^^,^ 39.

## Results

The means and standard deviations of the medial longitudinal arches for the acute and chronic PF groups were 0.15±0.05 and 0.17±0.09, respectively. With regards to the rearfoot angles, the values were 6.4±4.5 and 7.8±3.4 for the acute and chronic PF groups, respectively.

In the final regression model, the MLA index predicted a higher force-time integral over the forefoot for both PF groups and a higher maximum force over the forefoot in the chronic PF group (Table 2). However, the MLA index could not predict any loading rate variables for either PF group. The rearfoot valgus alignment predicted a higher maximum force over the rearfoot for both PF groups, in addition to predicting higher loading rates (20-80% and 0-100%) and higher force-time integrals over the rearfoot in the chronic PF group (Table 2).

**Table 2:** - The multiple regression models of the longitudinal plantar arch index (MLA) and rearfoot valgus alignment (REARFOOT) to predict the biomechanical dependent variables of runners with plantar fasciitis (PF): acute and chronic

Variables	Group	Beta Coefficient	Standard deviation	t	p&	Equation*	R, R^2^
Force-Time Integral (N.s) (FTIF)	Acute PF Chronic PF	0.350 0.165	0.16 0.12	3.0 3.2	0.031 0.020	FTIF=0.211+0.350*^MLA^ FTIF=6.096+0.165*^MLA^	*r* =0.35; *R* ^2^=0.15 *r* =0.41; *R* ^2^=0.17
Maximal Force forefoot (N) (MFF)	Chronic PF	1.850	0.81	2.2	0.043	MFF=1.580+1.850*^MLA^	*r* =0.59; *R* ^2^=0.35
Maximal Force rearfoot (N) (MFR)	Acute PF Chronic PF	2.012 0.056	0.17 0.02	2.8 2.7	0.048 0.017	MFR=1.400+2.012*^REARFOOT^ MFR=2.140+0.056*^REARFOOT^	*r* =0.42; *R* ^2^=0.18 *r* =0.67; *R* ^2^=0.45
Force-Time Integral rearfoot (N.s) (FTIR)	Chronic PF	0.103	0.12	3.8	0.041	FTIR=2.840+0.103*^REARFOOT^	*r* =0.41; *R* ^2^=0.17
Loading rate 20-80% Chronic PF (BW.s^–1^)		0.278	0.01	1.6	0.013	Loading rate (20-80%) =0.645+0.278*^REARFOOT^	*r* =0.44, *R* ^2^=0.19
Loading rate 0-100% Chronic PF (BW.s^–1^)		1.238	0.14	1.8	0.012	Loading rate (0-100%) =7.54+1.238*^REARFOOT^	*r* =0.63, *R* ^2^=0.40

Acronyms: PF: Plantar Fasciitis; MLA: Medial Longitudinal Arch; REARFOOT: rearfoot valgus alignment; FTIF: Force-Time Integral; MFF: Maximal Force Forefoot; MFR: Maximal Force Rearfoot; FTIR: Force-Time Integral Rearfoot. & p-value of the multiple regression analyses. *Equations of the multiple regression analyses. The t-value and resulting p-value are used to test the hypothesis that the intercept is equal to 0.

High intra-examiner (pre: 0.178±0.09 cm; post: 0.177±0.08 cm; SEM=0.10; ICC=0.92; 95% CI=0.84-0.78) and inter-examiner (examiner 1: 0.178±0.09 cm, SEP=0.02; examiner 2: 0.186±0.05 cm, SEP=0.01; ICC=0.90 95% CI=0.89-0.80) reliability levels were found for the MLA index. The rearfoot alignment reliability levels were also high for the intra-examiner (pre: 6.7±2.3 degrees; post: 6.5±2.7 degrees; SEM=0.7; ICC=0.95; 95%CI=0.87-0.78) and inter-examiner (examiner 1: 6.7±2.3 degrees, SEP=0.7; examiner 2: 6.3±2.9 degrees, SEP=0.9; ICC=0.90; 95% CI=0.83-0.77) measurements.

## Discussion

To the best of our knowledge, this is the first study to investigate the relationship between static foot alignment and plantar pressure patterns in runners with different stages of PF, as main risk factors for PF^23^^,^ 26. In contrast to what we hypothesized, an elevated MLA predicted higher plantar loads (higher force-time integral) over the forefoot while running in both PF groups and a higher maximum force over the forefoot in chronic PF (not only in runners with acute PF). This last finding suggests that higher loads over the forefoot, particularly during the propulsion of running, are strongly associated with elevated MLAs in runners with PF. This combination of the arch structure and loading pattern could indirectly result in higher tension in the plantar fascia around the metatarsal heads^40^, contributing to the progression of PF, regardless of its phase. Another important result allowed us to confirm our second hypothesis, which states that static valgus rearfoot alignment predicts higher plantar loads (higher maximum force and force-time integral) and higher loading rates (20-80% and 0-100%) over the rearfoot in both groups of PF.

During gait, some authors observed positive correlations between elevated MLAs and higher forefoot impulses^41^, and between elevated MLAs with foot pain symptoms and higher pressure-time integrals over the forefoot^42^. Although the present study evaluated the relationship between the foot posture and pressure while running, our results are similar to the gait findings^41^^,^ 42, demonstrating the predictive association between an elevated MLA and increased plantar load over the forefoot. One possible explanation for the positive relationship between an elevated MLA and higher maximum force over the forefoot in the group with chronic PF may be attributed to the foot's passive tissue and muscle changes^30^^,^ 31, such as reduced thickness of the plantar fascia and atrophy of the intrinsic musculature^43^, which, in turn, would affect the function of the MLA while running. In this study, the altered function resulted in reduced loads over the rearfoot and higher loads over the forefoot.

Other studies have reported positive correlations between elevated MLAs and higher loading rates, measured by force plates in healthy individuals while running^32^^,^ 33. Contrarily, Ramskov et al.^44^ observed that the static foot posture, quantified by the Foot Posture Index, did not seem to affect the risk of injury among novice runners. However, these results should be interpreted with caution due to the small sample size. The advantage of our study was to prove that static foot alignment (elevated MLA) in runners with PF, a more prevalent injury in runners^8^^,^ 9, predicted a higher plantar load over the forefoot while running, depending on the stage of PF (acute or chronic). However, the absence of a control group is one limitation of the present study.

One important finding was the non-confirmed relationship between an elevated MLA and higher plantar loads, or loading rates, while running, over the rearfoot in runners with PF (acute and chronic phases), since the rearfoot area is the region most associated with the physiopathology and etiology of PF^7^. A possible explanation for this finding is that runners in the acute phase of PF, with inflammation present in the calcaneal region, may have increased thickness of the plantar fascia (perifascial and cellular fluid collection), resulting in the reduced capacity of this tissue to support mechanical loads over the rearfoot area^23^^,^ 45. The consequence of this reduced capacity of the attenuating loads in the plantar fascia can lead to the adoption of an antalgic strategy to reduce the plantar load over the rearfoot, resulting in an increase in the plantar load over the forefoot. These results were also observed in studies that evaluated the gait task^7^^,^ 33. In addition, Sullivan et al.^46^ showed that people with heel pain had reduced maximum force, peak pressures, and force-time integrals over the heel while walking.

We hypothesized that the structure of an elevated MLA and valgus rearfoot alignment would predict higher plantar loads and loading rates over the rearfoot in both groups of runners. However, only the static valgus rearfoot alignment was a significant predictor of the maximum force and the force-time integrals, as well as the higher loading rates over the rearfoot in both PF groups. Our results agreed with the study by Pohl et al.^4^, who found increases in the vertical force in female runners with histories of PF, compared with control runners, although these authors did not show a statistical model demonstrating a direct relationship between these two parameters (MLA and plantar loads).

Higher and repetitive plantar loads over the rearfoot while running, due to valgus rearfoot alignment, can indirectly induce the tensile force and micro-failure of the plantar fascia throughout the medial calcaneal tuberosity while running^23^, contributing to the progression of PF. Lee and Hertel^26^ showed that valgus rearfoot alignment was a significant predictor of the peak and pressure-time integrals over the medial rearfoot and midfoot in healthy runners running on a treadmill. In the current study, we confirmed that runners with PF presented positive relationships between the valgus rearfoot alignment and maximum force, time-integral forces, and load rates over the rearfoot while running in a natural environment. Therefore, we can conclude that controlling the valgus alignment of the rearfoot may help prevent PF (acute and chronic). These findings may help health care professionals to choose more appropriate mechanical treatment strategies for runners with PF, such as orthoses, insoles, and physical therapy interventions, for better controlling rearfoot valgus and reducing the loading rates over the rearfoot.

One of the limitations of this study was that the loading rates were estimated using equipment with a maximal sampling rate of 100 Hz. We suggest that further studies examine the rearfoot valgus alignment and MLA dynamically to clearly elucidate the underlying mechanism of the increased maximum force, time-integral force, and loading rate over the rearfoot, as well as the maximum pressure and time-integral pressure over the forefoot, and relationships between these clinical measurements of the foot.

## Conclusions

An elevated MLA was shown to predict higher plantar loads over the forefoot in both groups of runners with PF (acute and chronic). The rearfoot valgus alignment was determined to be a good clinical measurement for predicting increases in the maximum force, force-time integral, and loading rates over the rearfoot in runners in both acute and chronic PF. Both clinical measurements showed relationships with the plantar loads and may contribute to the progression of PF, regardless of its phase.
